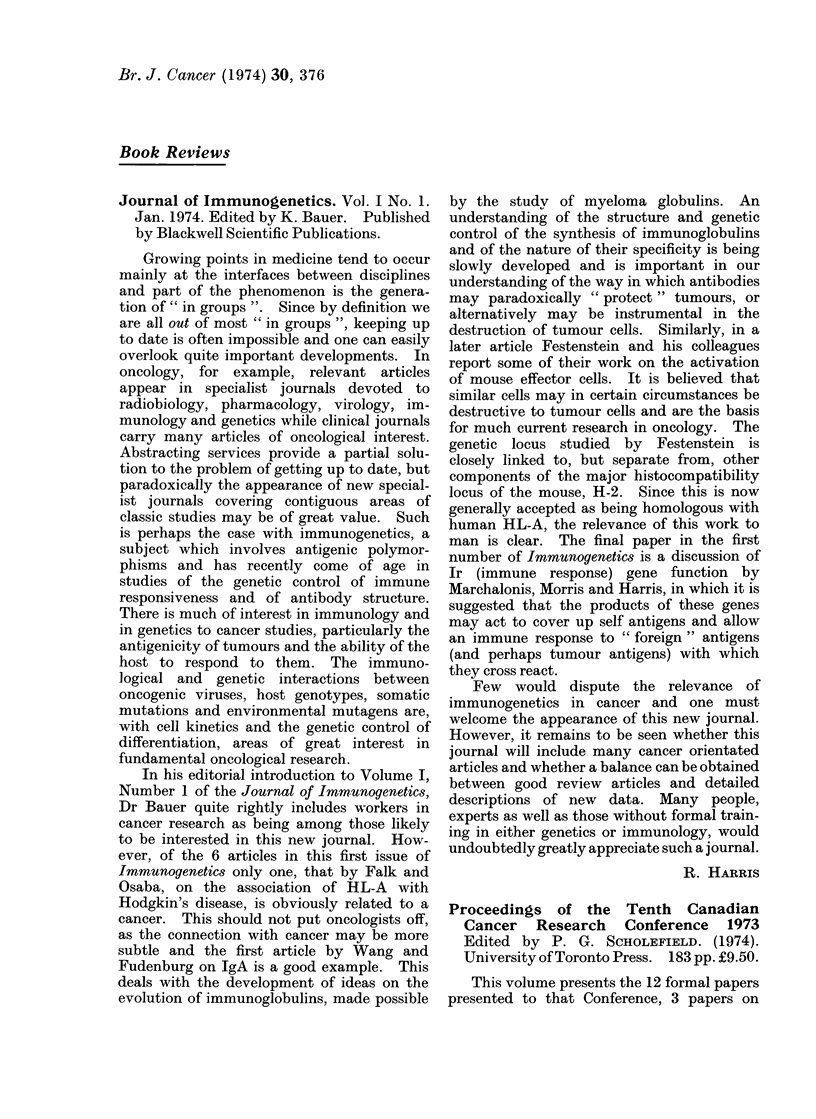# Journal of Immunogenetics

**Published:** 1974-10

**Authors:** R. Harris


*Br. J. Cancer* (1974) **30**, 376

### *Book Reviews*

**Journal of Immunogenetics.** Vol. I No. 1. Jan. 1974. Edited by K. Bauer. Published by Blackwell Scientific Publications.

Growing points in medicine tend to occur mainly at the interfaces between disciplines and part of the phenomenon is the generation of " in groups ". Since by definition we are all *out* of most " in groups ", keeping up to date is often impossible and one can easily overlook quite important developments. In oncology, for example, relevant articles appear in specialist journals devoted to radiobiology, pharmacology, virology, immunology and genetics while clinical journals carry many articles of oncological interest. Abstracting services provide a partial solution to the problem of getting up to date, but paradoxically the appearance of new specialist journals covering contiguous areas of classic studies may be of great value. Such is perhaps the case with immunogenetics, a subject which involves antigenic polymorphisms and has recently come of age in studies of the genetic control of immune responsiveness and of antibody structure. There is much of interest in immunology and in genetics to cancer studies, particularly the antigenicity of tumours and the ability of the host to respond to them. The immunological and genetic interactions between oncogenic viruses, host genotypes, somatic mutations and environmental mutagens are, with cell kinetics and the genetic control of differentiation, areas of great interest in fundamental oncological research.

In his editorial introduction to Volume I, Number 1 of the *Journal of Immunogenetics*, Dr Bauer quite rightly includes workers in cancer research as being among those likely to be interested in this new journal. However, of the 6 articles in this first issue of *Immunogenetics* only one, that by Falk and Osaba, on the association of HL-A with Hodgkin's disease, is obviously related to a cancer. This should not put oncologists off, as the connection with cancer may be more subtle and the first article by Wang and Fudenburg on IgA is a good example. This deals with the development of ideas on the evolution of immunoglobulins, made possible by the study of myeloma globulins. An understanding of the structure and genetic control of the synthesis of immunoglobulins and of the nature of their specificity is being slowly developed and is important in our understanding of the way in which antibodies may paradoxically " protect " tumours, or alternatively may be instrumental in the destruction of tumour cells. Similarly, in a later article Festenstein and his colleagues report some of their work on the activation of mouse effector cells. It is believed that similar cells may in certain circumstances be destructive to tumour cells and are the basis for much current research in oncology. The genetic locus studied by Festenstein is closely linked to, but separate from, other components of the major histocompatibility locus of the mouse, H-2. Since this is now generally accepted as being homologous with human HL-A, the relevance of this work to man is clear. The final paper in the first number of *Immunogenetics* is a discussion of Ir (immune response) gene function by Marchalonis, Morris and Harris, in which it is suggested that the products of these genes may act to cover up self antigens and allow an immune response to " foreign " antigens (and perhaps tumour antigens) with which they cross react.

Few would dispute the relevance of immunogenetics in cancer and one must welcome the appearance of this new journal. However, it remains to be seen whether this journal will include many cancer orientated articles and whether a balance can be obtained between good review articles and detailed descriptions of new data. Many people, experts as well as those without formal training in either genetics or immunology, would undoubtedly greatly appreciate such a journal.

R. HARRIS